# Azithromycin Prescribing by Respiratory Pediatricians in Australia and New Zealand for Chronic Wet Cough: A Questionnaire-Based Survey

**DOI:** 10.3389/fped.2020.00519

**Published:** 2020-09-02

**Authors:** Naomi Chellew, Anne B. Chang, Keith Grimwood

**Affiliations:** ^1^Department of Paediatrics, Gold Coast Health, Southport, QLD, Australia; ^2^Department of Respiratory and Sleep Medicine, Queensland Children's Hospital and Australian Centre for Health Services Innovation, Queensland University of Technology, Brisbane, QLD, Australia; ^3^Child Health Division, Menzies School of Health Research, Charles Darwin University, Darwin, NT, Australia; ^4^School of Medicine and Menzies Health Institute Queensland, Griffith University, Gold Coast Campus, Southport, QLD, Australia

**Keywords:** azithromycin, chronic cough, protracted bacterial bronchitis, chronic suppurative lung disease, bronchiectasis

## Abstract

**Aims:** To determine how respiratory pediatricians across Australia and New Zealand prescribe azithromycin for children with chronic wet cough, including recurrent protracted bacterial bronchitis, chronic suppurative lung disease (CSLD) and bronchiectasis.

**Methods:** A prospective web-based questionnaire was emailed to members of the Pediatric Special Interest Group of the Thoracic Society of Australia and New Zealand (TSANZ) between April and May 2018. It comprised eight demographic and 15 clinically focused questions.

**Results:** Of the 73 respiratory pediatricians listed across Australia and New Zealand, 29 (40%) responded and all prescribed azithromycin for chronic wet cough. Twelve (41%) indicated that they would consider prescribing a short-course (2–4 weeks) of azithromycin for children with a chronic wet cough. Although most respondents reported prescribing long-term (>4-weeks) azithromycin for either CSLD (*n* = 23, 79%) or bronchiectasis (*n* = 24, 83%), only nine (31%) respondents would commence treatment if in the previous 12-months these children experienced three non-hospitalized exacerbations and just 12 (41%) would do so if there had been two hospitalisations for severe exacerbations during the same period in accordance with the TSANZ national guidelines. A lower threshold for prescribing azithromycin was described for Indigenous children or if co-morbidities were present. None prescribed azithromycin for >24-months. Macrolide-resistance was reported in *Streptococcus pneumoniae* and *Staphylococcus aureus*.

**Conclusion:** Although Australian and New Zealand respiratory pediatricians in this survey prescribed azithromycin for chronic wet cough most often in children with either CSLD or bronchiectasis, many did so outside the current national guidelines. Reasons for this need exploring.

## Introduction

Chronic (>4-weeks) wet cough accompanied by bacterial infection and neutrophilic infiltration of the lower airways are common clinical features of endobronchial suppuration present in protracted bacterial bronchitis (PBB), recurrent protracted bacterial bronchitis (rPBB), chronic suppurative lung disease (CSLD), and bronchiectasis. They form a continuum of increasing severity and are being recognized increasingly in children worldwide, (1) especially among disadvantaged Indigenous populations (2). As early and effective therapy influences prognosis and quality of life in children with these chronic pulmonary disorders, it is essential that treating physicians are aware of how to optimize their management (2, 3). Despite antibiotics being considered critical for treating conditions associated with a chronic wet or productive cough, there is limited clinical trial evidence informing optimal antibiotic choice and dosing regimens, or when and in whom to commence long-term antibiotics and how to conduct ongoing monitoring of their effectiveness.

The macrolide, azithromycin, is a potentially attractive option for treating patients with a chronic wet cough. It is well-tolerated, has good oral bioavailability and a prolonged half-life allowing convenient once-daily or even one-to three times weekly dosing (4). Importantly, azithromycin is active against most strains of *Haemophilus influenzae, Moraxella catarrhalis, Streptococcus pneumoniae* and *Staphylococcus aureus*, pathogens commonly identified in patients with PBB, rPBB, CSLD and bronchiectasis (4, 5). Furthermore, azithromycin has immunomodulatory properties that may limit airway wall damage secondary to infection and inflammation (4). However, azithromycin also induces macrolide resistance amongst commensal and pathogenic organisms, which may have adverse effects at a personal health and population-level (6).

National [Thoracic Society of Australia and New Zealand [TSANZ]] guidelines for managing chronic cough, (7) as well as recent counterpart European and North American guidelines that have had input from Australian and New Zealand pediatricians (8, 9), recommend 2–4 weeks of antibiotic therapy (usually oral amoxicillin-clavulanate) for treating PBB, with macrolides reserved for those with penicillin allergy. For adults and children with CSLD and bronchiectasis, recent TSANZ guidelines exist, but there are no similar European or North American published guidelines for children with bronchiectasis. The TSANZ guidelines recommend that macrolides are used only as long-term (>4-weeks) oral antibiotics and for a limited period of 12–24 months in those experiencing three or more exacerbations and/or at least two respiratory hospitalisations in the previous year (10). For children, these recommendations were based on a single placebo-controlled, randomized controlled trial (RCT) of azithromycin in Indigenous Australian and New Zealand children, which showed azithromycin administered in a single weekly dose for up to 24-months resulted in a 50% reduction in acute exacerbations compared with participants given placebo (11). Those prescribed long-term macrolides must be without non-tuberculous mycobacterial (NTM) infection and in adults lack risk factors for QTc interval prolongation with a normal electrocardiogram (10). As the optimal duration of long-term therapy is unknown, some experts recommend a more pragmatic approach if there is a seasonal component to the exacerbation frequency, limiting treatment to the months of the year where exacerbations have been most frequent (12).

Despite these recommendations, amongst the macrolides, azithromycin has become a popular choice for treating children with a chronic wet cough in some regions of Australia where more than 50% of those undergoing bronchoscopy for this symptom have received this antibiotic in the preceding 14-days (13). This high use of macrolides has implications for community antimicrobial resistance (14).

Against this background we sought to determine the prescribing of azithromycin by Australian and New Zealand respiratory pediatricians for pulmonary disorders associated with a chronic wet cough and how this aligns with the national management guidelines.

## Methods

A pilot web-based questionnaire was designed and sent to two respiratory pediatricians and to 11 general pediatricians practicing on the Gold Coast, South-East Queensland, Australia. Following additional amendments, the SurveyMonkey incorporated questionnaire (SurveyMonkey Australia Pty Limited, Sydney, Australia) was then electronically distributed via email in April 2018 to the 73 members of the TSANZ Pediatric Special Interest Group. The email was re-distributed twice again over the following 6-weeks.

Clinicians were presented with 23 questions in the survey; eight questions gathered demographic information and explored the nature of their clinical practice, whilst the remaining 15 questions related to prescribing azithromycin (Appendix S1, Supporting Information). The Gold Coast Hospital and Health Services Human Research Ethics Committee approved this study (HREC/18/QGC/40).

### Definitions

We used the following definitions for the survey:

**PBB**: an isolated chronic (>4-weeks) wet or moist cough without specific pointers to an underlying cause and which resolves after a 2–4 weeks course of oral antibiotics (8).

**rPBB**: >3 episodes of PBB in a 12-month period (15).

**CSLD**: where symptoms and signs of bronchiectasis are present, but there is no high-resolution computed-tomography (HRCT) scan evidence of this diagnosis (16).

**Bronchiectasis**: characterized by chronic wet cough with a variable response to antibiotics, frequent pulmonary exacerbations and HRCT scan evidence of one or more abnormally dilated bronchi (17).

## Results

**Figure 1:**
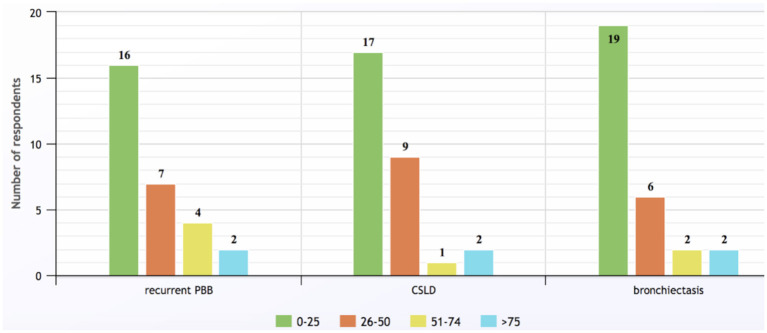
Numbers of children with recurrent protracted bacterial bronchitis, chronic suppurative lung disease, and bronchiectasis seen by survey respondents annually (response to Q6).

Overall, 29/73 (40%, 15 males) physicians responded to the questionnaire. Their modal decade of medical graduation was 2000–2009. Respondents came from New Zealand and all Australian states/territories other than Tasmania, with the greatest number practicing in Queensland (9/29, 31%). Eighteen (62%) worked solely in public hospitals (including two who practiced in two states), 11 (38%) had both private and public hospital sessions and two (7%) also worked in community clinics. Annually, most physicians saw <25 cases of rPBB (*n* = 16, 55%), CSLD (*n* = 17, 59%) and bronchiectasis (*n* = 19, 66% respondents), but two from tertiary pediatric hospitals managed individually >75 patients for each of the three conditions (Figure 1).

Azithromycin was prescribed by all 29 respondents with 14 (48%) using this antibiotic to treat >25 children for a chronic wet cough in the previous 12-months. Although not a recommended first-line antibiotic for PBB, 12 (41%) would consider using it in a short course (2–4 weeks) to treat a chronic wet cough. Other indications for contemplating short courses of azithromycin were rPBB (*n* = 9, 31%) and non-severe exacerbations in children with CSLD (*n* = 10, 34%), bronchiectasis (*n* = 9, 31%) or chronic aspiration (*n* = 5, 17%). However, only two (7%) prescribed azithromycin as first-line treatment for these conditions.

In contrast, most respondents considered long-term azithromycin (>4-weeks) for CSLD (*n* = 23, 79%) and bronchiectasis (*n* = 24, 83%). Interestingly, nine (31%) also used longer courses of azithromycin for PBB. Overall, just nine (31%) respondents would follow TSANZ guidelines (10) by prescribing long-term azithromycin to children with either CSLD or bronchiectasis and who had experienced three non-hospitalized exacerbations in the previous 12-months, while only 12 (41%) would initiate azithromycin following two hospitalisations for these children during the same 12-month period. Two respiratory pediatricians confined prescribing long-term azithromycin to their cystic fibrosis (CF) patients and another prescribed the antibiotic routinely for all patients with bronchiectasis, irrespective of their exacerbation history in the last 12-months.

Prior to commencing azithromycin, respondents ordered a range of investigations, the most frequent being sputum bacteriology (*n* = 27, 93%) and spirometry (*n* = 23, 79%) in children able to expectorate and perform lung function tests, respectively. However, only 17 (58%) requested NTM cultures when collecting sputum. Although not specifically recommended for children in the TSANZ guidelines, (10) 10 (35%) performed a baseline electrocardiogram and two (7%) checked hearing before commencing long-term azithromycin. Only one respiratory pediatrician did not order any investigations, while another two rarely commenced azithromycin without performing bronchoscopy.

**Figure 2:**
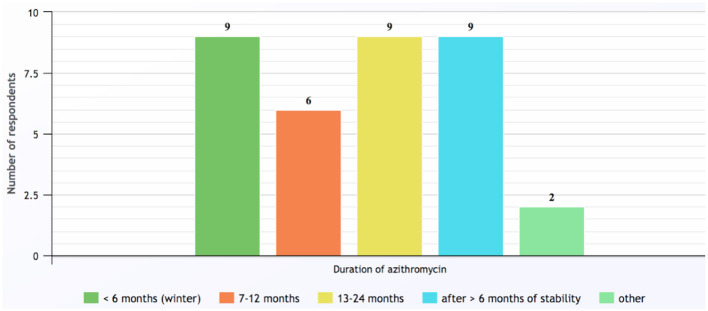
Duration of long-term azithromycin courses prescribed by respondents for children with chronic suppurative lung disease and bronchiectasis. Note: category numbers are not mutually exclusive. Other responses included: Azithromycin prescribed only for cystic fibrosis patients; and duration determined by patient's age and previous response following cessation of azithromycin (response to Q16).

Contraindications for commencing azithromycin identified by the responding respiratory pediatricians included hypersensitivity to macrolides (*n* = 27, 93%), NTM infection (*n* = 21, 72%) and abnormal liver function tests (*n* = 15, 52%). Once the decision was made to begin long-term azithromycin, the most common dose prescribed was 10 mg/kg (up to 250 mg) three-times a week, which was used by 90% of respiratory pediatricians. Other dosing regimens employed included 500 mg three-times a week (17%), 5 mg/kg (up to 250 mg) daily (14%), and weekly dosing of 30 mg/kg (14%). The duration of azithromycin long-term therapy was <6-months if prescribed only during the winter season, otherwise courses were for periods of 6–24 months or until clinically stable and having had no exacerbations in the last 6-months (Figure 2). While taking azithromycin, most patients were reviewed by their respiratory pediatrician every 3-months (*n* = 22, 76%) and 26 (90%) reported monitoring lung function using spirometry and 22 (76%) performed sputum cultures in those able to perform either of these tests, respectively. Another eight (28%) monitored liver function tests.

Reasons given by respiratory pediatricians for discontinuing azithromycin prematurely included if children experienced severe gastrointestinal adverse effects (*n* = 21, 72%), there was no improvement after 3–6 months (*n* = 19, 66%,) if NTM was cultured in sputum (*n* = 19, 66%), when macrolide-resistant pathogens were detected (*n* = 15, 52%) or if there was poor adherence with the medication (*n* = 3, 10%).

**Figure 3:**
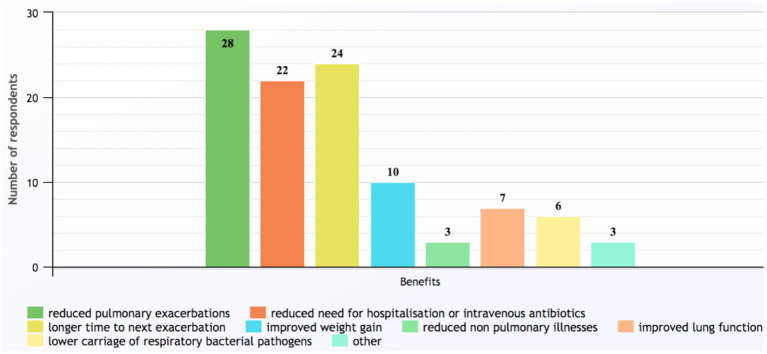
Benefits of long-term azithromycin reported by survey respondents. Other responses included: unsure; improved asthma control; and better quality of life (response to Q20).

The most common benefit found in children receiving long-term azithromycin was reduced pulmonary exacerbations, which was reported by 28 (97%) respondents (Figure 3). In contrast, most (*n* = 17, 58%) were uncertain how long the beneficial effects persisted after ceasing the antibiotic, while seven (24%) believed beneficial effects lasted for another 3–12 months.

Macrolide-resistant pathogens were reported by 18 (62%) respiratory pediatricians in children taking azithromycin. The remaining 11 did not observe or test for macrolide resistance in sputum-cultured pathogens. Macrolide-resistance was reported in *H. influenzae* by eight (28%) respondents, in *S. pneumoniae* and *S. aureus* each by another six (21%) and in *M. catarrhalis* by four physicians (14%).

Finally, 18 (62%) respondents were more likely to prescribe azithromycin for Indigenous children, while 15 (52%) also reported being more likely to prescribe the antibiotic for those with major underlying co-morbidities (e.g., congenital heart disease, neuromuscular disorder, cerebral palsy, acquired brain injury, congenital immune deficiency or immunosuppression). One reason provided for preferring azithromycin was the convenience of once-daily over multiple-daily dosing for the antibiotic when treating PBB, rPBB, and non-severe exacerbations of CSLD and bronchiectasis.

## Discussion

The survey identified the current prescribing practices of respiratory pediatricians across Australia and New Zealand for azithromycin in children with a chronic wet cough. We found that azithromycin was used widely. However, adherence to current national recommendations and guidelines were not always followed closely.

Areas of current practice of respiratory pediatricians in accordance with the TSANZ recommendations included 93% not using azithromycin as first-line treatment for either isolated chronic wet cough associated with PBB and rPBB (7) or for non-severe respiratory exacerbations of CSLD or bronchiectasis (10). Since completing the current survey, two RCTs involving azithromycin for treating non-severe exacerbations of bronchiectasis in children have been published (18, 19). BEST-1 was a multi-centre, three-arm, double-dummy, placebo-controlled RCT conducted in Australia and New Zealand (18). Oral amoxicillin-clavulanate was significantly superior to placebo at achieving symptom resolution after 14-days treatment and while those receiving oral azithromycin also reached the traditional statistical significance level (compared to placebo, p=0.042), the *a-priori* defined significance level (*p* < 0.0245) was not achieved. Furthermore, in BEST-2, a double-blind, double-dummy RCT also conducted in centers across Australia and New Zealand, oral azithromycin was non-inferior within a 20% margin to oral amoxicillin-clavulanate after 21-days treatment (19). However, episodes responding to treatment took on average 4-days longer to resolve in those receiving azithromycin than children allocated to amoxicillin-clavulanate, a statistically significant result. In both studies macrolide-resistant pathogens emerged during treatment with azithromycin, but not with amoxicillin-clavulanate (18, 19). Taken together, oral amoxicillin-clavulanate should remain the antibiotic of first-choice for treating PBB, rPBB, and non-severe exacerbations of CSLD and bronchiectasis, with azithromycin reserved for those with genuine penicillin allergy or where adherence is problematic.

In contrast, with most respondents avoiding short-courses of azithromycin for first-line treatment, almost one-third consider using longer azithromycin courses (>4-weeks) to treat isolated chronic cough of PBB or rPBB. This practice highlights differences between Australian (7) and British (20) guidelines where the latter advocate at least 4–6 weeks treatment. However, unlike the Australian and other international PBB guidelines, the British recommendation is not based upon prospective cohort or trial data and this is an important knowledge gap that requires addressing (7–9, 20).

When prescribing long-term azithromycin, respondents adhered to TSANZ recommendations (10) with none using courses >24-months. Consistent with some expert recommendations, (12) long-term azithromycin was prescribed by almost one-third of respiratory pediatricians for just the winter season. This practice highlights that the optimal duration for long-term azithromycin is uncertain and more trials are needed. For example, examination of our placebo-controlled RCT of long-term azithromycin in children with CSLD and bronchiectasis indicated that the maximal benefit from the antibiotic occurred between 4 and 15-months of therapy, (11) while in children with CF, clinical benefits may disappear after just 12-months of treatment (21).

Our survey also identified practices that deviated from the current TSANZ guidelines for long-term azithromycin in children with CSLD and bronchiectasis. Only one-third of respondents prescribed azithromycin for children experiencing three exacerbations and just 41% of respondents would do so for those with two respiratory hospitalisations in the previous year. Moreover, before commencing azithromycin <60% requested NTM cultures from those capable of producing sputum. Similar disparities between TSANZ guideline recommendations and actual clinical practice were also observed recently in Australian adults with bronchiectasis (22). Reassuringly, data from children with CF indicate that unlike in the elderly, azithromycin in children was not associated with sensorineural hearing loss or QTc interval prolongation (23). Azithromycin induces antibiotic resistance, which is an emerging public health threat (6). Overall, 14–28% reported macrolide resistance in *H. influenzae, S. pneumoniae, M. catarrhalis* and *S. aureus*. The increased incidence of macrolide-resistance in *S. pneumonia* and *S. aureus* following azithromycin is well-documented in Australian children with CSLD and bronchiectasis (11, 18, 19, 24). However, it is difficult to interpret resistance reported for *H. influenzae* and *M. catarrhalis* as these organisms decline with azithromycin and resistance is uncommon, even in regions of high macrolide use, such as the Northern Territory of Australia (11, 24).

Guidelines seek to decrease the gap between research and current practice and to reduce inappropriate variability in practice. However, the use of guidelines is highly variable. Those that improve patient outcomes and are based on good evidence are more likely to be used and adopted into clinical practice (25). Our study did not evaluate why some respondents did not adhere to the relevant guideline. Possible reasons include insufficient evidence to support a recommendation, lack of awareness of the guidelines, conflicting data, and inadequate guideline dissemination and implementation (26). While the TSANZ guidelines were transparent and based on systematic reviews, they were limited by the large knowledge gaps involving when, in whom and how to prescribe azithromycin for children with chronic wet cough (7–10).

The global tendency to overuse antibiotics in general, especially for acute respiratory infections, is well-documented and that specifically for azithromycin has been reported (27). In non-acute respiratory diseases, azithromycin is being trialed increasingly for many non-infectious conditions, including asthma, (28) refractory chronic cough, (29) and pulmonary fibrosis (30). In this context and the lack of RCTs on the managing of PBB and recurrent PBB, (8, 9) the use of azithromycin outside current evidence-based guidelines is not surprising. However, given the implications for community antimicrobial resistance when macrolide use is high, (14) survey data like our study is arguably important.

Our study is the first examining the use of azithromycin for respiratory conditions not related to acute respiratory infections (PubMed search on 30th May 2020). As we are based in Australasia, we surveyed Australian and New Zealand respiratory pediatrician prescribing practice for azithromycin. Despite our study's important findings, it has substantial limitations. Firstly, our response rate across Australia and New Zealand was just 40%, and thus the data might not represent the practice of all respiratory pediatricians because of the 60% non-response bias. However, this rate is similar to other surveys where the denominator is known; such as a recent survey on clinicians' practice in managing pediatric *Staphylococcus aureus* bacteraemia (31). Further, the responses are still valid in that they reflect the practice of a sizeable minority of prescribers. Secondly, some questions required data from the last 12-months, introducing the possibility of recall bias with some responses. Thirdly, this survey involved respiratory pediatricians from Australia and New Zealand where relatively high rates of PBB (32) and bronchiectasis (17) are reported. Consequently, the results may not be generalisable to other populations. Moreover, the practice of experienced respiratory pediatricians who regularly review children with chronic cough may not be representative of other clinicians who can also access the TSANZ guidelines. Finally, the answers provided may not reflect actual practice, and some respondents may also have interpreted certain questions as testing knowledge, rather than reporting their clinical practice and observations. Since answers to the survey were anonymous to help reporting clinical practice more accurately this also prevented clarifying how questions were understood.

In conclusion, respiratory pediatricians participating in this survey from Australia and New Zealand commonly prescribed azithromycin for children with a chronic wet cough. Although they adhered to some recommendations, many had prescription patterns outside the current TSANZ recommendations. Azithromycin prescribing for periods of 6–24 months should balance the benefits of reduced exacerbation frequency with inducing macrolide resistance, where the impact upon the host microbiome and personal and population health remains uncertain. This survey highlights the importance of closing knowledge gaps around whom, when and for how long azithromycin should be prescribed. Some of this has been addressed recently for acute exacerbations of CSLD and bronchiectasis, (16, 17, 19) which now needs to be reflected in updated evidence-based national guidelines. In parallel, increasing practitioner awareness and knowledge of appropriate azithromycin prescribing for children with chronic wet cough should continue as this is as important as developing and updating evidence-based guidelines.

## Data Availability Statement

All datasets presented in this study are included in the article/Supplementary Material.

## Ethics Statement

The Gold Coast Hospital and Health Services Human Research Ethics Committee approved this study (HREC/18/QGC/40).

## Author Contributions

NC, KG, and AC contributed to the development of the questionnaire. NC primarily wrote the manuscript. KG and AC were involved in the planning of the study, discussion of results, and finalization of the paper. All authors contributed to the article and approved the submitted version.

## Conflict of Interest

AC is funded by a NHMRC practitioner fellowship (APP1058213) and Queensland Children's Hospital Foundation (50286). KG and AC have received multiple NHMRC grants relating to the prevention and treatment of bronchiectasis and chronic cough. The remaining author declares that the research was conducted in the absence of any commercial or financial relationships that could be construed as a potential conflict of interest.
