# Supplementary material for: Azithromycin Prescribing by Respiratory Pediatricians in Australia and New Zealand for Chronic Wet Cough: A Questionnaire-Based Survey
Source: Front Pediatr. 2020 Sep 2;8:519. doi: 10.3389/fped.2020.00519 (PMC7492546; doi:10.3389/fped.2020.00519)
Supplement: Supplementary file 1 [file Data_Sheet_1.PDF]

Supplementary File

Here are the questions, unchanged, from the online survey, hosted on SurveyMonkey, publicised through the Thoracic Society of Australia and New Zealand Paediatric Special Interest Group. The survey was open from April to May 2018.

Survey title: Azithromycin prescribing by respiratory paediatricians in Australia and New Zealand for chronic wet cough: a questionnaire-based survey.

I am wanting to learn how the antibiotic, azithromycin, is being prescribed by paediatric respiratory specialists in Australia and New Zealand for children with symptoms of chronic cough and/or features of chronic suppurative lung disease or bronchiectasis. The first 8 questions of the survey are gathering demographic information and learning about the nature of your clinical practice, while the remaining 15 questions are about where, when and to whom you prescribe azithromycin.
I would really appreciate the 10 minutes you spend to complete this short survey.

Definitions used for the purpose of this survey:

- **Protracted bacterial bronchitis** (PBB) - an isolated chronic (>4 weeks) wet or moist cough without specific pointers to an underlying cause and which resolves after a 2-4 week course of oral antibiotics.

- **Recurrent PBB** - >3 episodes of PBB in a 12-month period.

- **Chronic suppurative lung disease** (CSLD) - where symptoms and signs of bronchiectasis are present, but HRCT scan evidence for this diagnosis is absent.

- **Bronchiectasis** - characterised by chronic wet cough with a variable response to antibiotics, frequent pulmonary exacerbations and HRCT scan evidence of one or more dilated bronchi.

'This survey is being conducted using SurveyMonkey, which is based in the United States of America. Information you provide on this survey will be transferred to SurveyMonkey's server in the United States of America. By completing the survey, you agree to this transfer.'

All data collected will be kept confidential.

All data collected will be kept confidential.

Question 1-8 refer to your demographics and clinical setting.

1.What year did you graduate from medical school?

○1960-1969 ○1970-1979 ○1980-1989 ○1990-1999 ○2000-2009

2. What is your gender?

○male ○female

3. What is your medical discipline?

- ○ specialist respiratory paediatrician (in respiratory)
- ○ specialist respiratory paediatrican within general paediatrics
- ○ general paediatrician with a special interest in respiratory medicine
- ○ other

4. What is the nature of your practice?

- ○ public hospital only
- ○ public and private
- ○ private only
- ○ community clinic
- ○ Other (please specify)

5. What state or country do you practice in?

- ○ Australian Capital Territory
- ○ New South Wales
- ○ Northern Territory
- ○ Queensland
- ○ South Australia
- ○ Tasmania
- ○ Victoria

○ Western Australia

○ New Zealand

6. How many patients approximately do you see annually with?

	0-25	26-50	51-74	>75
bronchiectasis unrelated to cystic fibrosis	○	○	○	○
chronic suppurative lung disease	○	○	○	○
recurrent (>3 episodes/yr) of protracted bacterial bronchitis	○	○	○	○

7. What conditions/circumstances would you consider prescribing azithromycin? (tick all that apply)

Short course (2-4 weeks)

○ Bronchiectasis

○ chronic suppurative lung disease (CSLD)

○ recurrent (>3 episodes/year) of protracted bacterial bronchitis

○ chronic (>4 weeks) of wet cough where protracted bacterial bronchitis is considered the most likely diagnosis

○ chronic aspiration (e.g. child with cerebral palsy, acquired brain injury or other neuromuscular disorders.)

○ Other (please specify)

Long Course (>4 weeks)

○ Bronchiectasis

○ chronic suppurative lung disease (CSLD)

○ recurrent (>3 episodes/year) of protracted bacterial bronchitis

○ chronic (>4 weeks) of wet cough where protracted bacterial bronchitis is considered the most likely diagnosis

○ chronic aspiration (e.g. child with cerebral palsy, acquired brain injury or other neuromuscular disorders.)

○ Other (please specify)

8. Approximately how many children have you prescribed azithromycin to in the last 12 months?

0-25 ○ 26-50 ○ 51-74 ○ >75 ○

The following questions (Q9-23) refer to long term (> 4weeks) therapy for patients with CSLD or bronchiectasis.

9. After how many <u>non-hospitalised</u> exacerbations of CSLD or bronchiectasis would you prescribe azithromycin?

○ 1 exacerbation in 12 months
○ 2 exacerbations in 12 months
○ 3 exacerbations in 12 months
○ 4 exacerbations in 12 months
○ 5 exacerbations in 12 months
○ Other (please specify)

10. After how many <u>hospitalisation</u> for CSLD or bronchiectasis would you prescribe azithromycin?

○ 1 hospitilisation in 12 months
○ 2 hospitalisation 12 months
○ 3 hospitalisation in 12 months
○ 4 hospitalisation 12 months
○ 5 hospitalisation in 12 months
○ Other (please specify)

11. Do you prescribe azithromycin as a first line treatment for non-severe (non-hospitalised) respiratory exacerbation in CSLD or bronchiectasis?

○yes ○no

12. Do you trial a long-term non-macrolide antibiotic (e.g. trimethoprim/ sulfamethoxazole for 3 months) prior to commencing azithromycin?

○ yes - usually (>75% of cases) ○yes - often (25-74% of cases)

○yes - sometimes (<25% of cases) ○no

13. What tests do you routinely perform prior to commencing azithromycin? (tick all that apply)

○C- reactive protein (CRP)

○Liver function test

○ Upper airway swabs (nasal swab or throat swab)

○Sputum (culture & sensitivty) [when age permits]

○Non- tuberculous mycobacteria sputum [when age permits]

○Spirometry [when age permits]

○ ECG

○Hearing test

○None

○other – please specify

14. What are your contraindications to prescribing azithromycin? (tick all that apply)

○history of immediate or delayed hypersensitivity reactions to macrolides

○abnormal liver function tests

○ microbiologic al evidence of non- tuberculous mycobacteria infection

○ *Pseudomonas aeruginosa* colonisation

○ abnormal for- age hearing

○ QTc prolongation or risk factors for QTc prolongation

○ other (please specify)

15. What is the prophylactic dose of azithromycin you use? (tick all that apply)

○ 5mg/kg [up to 250mg] daily

○ 10mg/kg [up to 250mg three] times a week

○ 500mg three times a week

○ weekly (30mg/kg)

○ Other (please specify)

16. If the child has had no exacerbations since starting long-term azithromycin, when would you consider ceasing it? (tick all that apply)

○ < 6 months (e.g. after the winter season)

○ 7-12 months

○ 13-24 months

○ 25-36 months

○ after a period of stability of > 6 months

○ Other (please specify)

17. What are the major reasons for you to discontinue azithromycin? (tick all that apply)

- ○ no clinical improvement after 3-6 months
- ○ no clinical improvement after 7- 12 months
- ○ 4+ hospital inpatient managed pulmonary exacerbations in 12 months
- ○ gastrointestinal side effects
- ○ patient has been on medication for 2 years
- ○ detection of macrolide e-resistant bacterial pathogens on respiratory cultures
- ○ growth of non- tuberculous mycobacterium in sputum
- ○ period of stability > 6 months
- ○ Other (please specify)

18. What monitoring do you perform whilst the child is on azithromycin? (tick all that apply)

- ○ liver function test
- ○ respiratory cultures (testing for antimicrobial resistance to common respiratory pathogens.)
- ○ sputum microbiology (e.g. annual non-tuberculous mycobacteria cultures on those able to produce sputum)
- ○ spirometry (if age permits)
- ○ hearing test
- ○ none
- ○ Other (please specify)

19. What macrolide resistant pathogens do you find in children taking azithromycin? (tick all that apply)

○ *Streptococcus pneumoniae*

○ *Staphylococcus aureus*

○ *Moraxella catarrhalis*

○ non- tuberculous mycobacteria

○ *Haemophillus influenzae*

○ No evidence seen of resistance when tested

○ I do NOT test for resistant bacteria

○ Other (please specify)

20. What benefits do you see in the children on azithromycin? (tick all that apply)

○ reduced pulmonary exacerbations

○ reduced need for hospitalisation or intravenous antibiotics

○ longer time to next exacerbation

○ improved weight gain or body mass index

○ reduced non- pulmonary illnesses

○ improved lung function

○ lower carriage of respiratory bacterial pathogens e.g. *H.influenzae* or *M.catarrhalis* (if monitored)

○ Other (please specify)

21. Do you have a lower threshold for prescribing azithromycin in any of the following circumstances? (tick all that apply)

○ Indigenous (Aboriginal, Torres Straight Islander, South Sea Islander, Maori or Pasifica)

○ remote and rural community

○ younger age < 5 years

○ underlying co-morbidities or disorders (e.g. congenital heart disease, neuromuscular disorder, cerebral palsy, acquired brain injury, congenital immune deficiency or immunosuppression)

○ Other (please specify)

22. How often do you review the child whilst they are on azithromycin?

○ every 6 weeks

○ every 3 months

○ every 6 months

○ once a year

○ no follow up

○ Other (please specify)

23. How long after you cease azithromycin do you find the beneficial effects persist?

○ 3-6 months

○ 7-12 months

○ 13-24 months

○ 25-60 months

○ > 60 months

○ uncertain at this stage

○ other (please specify)
